# Restoration of femoral offset, rotation centers, limbs length equality of Chinese total hip arthroplasty patients

**DOI:** 10.12669/pjms.301.3635

**Published:** 2014

**Authors:** Yu-ping Liu, Yue-dong Hao

**Affiliations:** 1 Yu-ping Liu, Department of Trauma Orthopedics, Tengzhou Central People’s Hospital, Shandong, PR China.; 2 Yue-dong Hao, Department of Orthopedics, Huaian first people's hospital, Jiangsu, PR China.

**Keywords:** Femoral offset, Limbs length, Rotation centers, Total hip arthoplasty

## Abstract

***Objective: ***To explore the restoration of femoral offset, rotation centers, limbs length equality of Chinese total hip arthroplasty patients with careful preoperative surgical planning, the appropriate prosthesis and skillful manipulation combined with a variety of verification tests during the operation.

***Methods: ***There were 92 hips (from 92 patients) surgery was performed by the same surgeon using the posterlateral approach by careful preoperative surgical planning. Appropriate prosthesis was chosen determining the reasonable femur osteotomy location, skillful manipulation and paying attention to every detail combined with a variety of verification tests and preoperative measurements during the operation. We evaluated the offset and rotation centers of the healthy (not performed) side and the operated side, the preoperative and postoperative limbs length discrepancy and analyzed the change of femoral offset, rotation centers and limbs length discrepancy of THA patients by self-control.

***Results:*** We found that the preoperative and postoperative femoral offset was basically not changed, the postoperative rotation centers had a tendency to the medial and inferior of the original rotation centers, the limbs length discrepancy and Harris Hip Score (HHS) were improved much more than before.

***Conclusions:*** Careful preoperative surgical planning, the appropriate prosthesis and skillful manipulation combined with a variety of verification tests during the operation is significantly correlated to the remarkable radiological and clinical results of THA patients.

## METHODS

***Patients: ***From January 2002 to December 2007, a total of 92 primary THA patients with unilateral hip disease aged from 23 to 78 were performed by the same surgeon, using the Modular total hip prostheses (Link corporation, Germany, the cemented prostheses with SP-II or Classic, the cementless prostheses with Rebed and hybrid prostheses of these three). All patients were operated on using a posterlateral approach in each surgery with the anterior joint capsule and gluteus mdius muscle being preserved. The classification of the diseases of the 92 patients included femoral neck fracture, degenerative hip disease, and femur head necrosis and so on. All the patients were only performed in one limb. No Developmental Dysplasia Of The Hip or other congenital hip disease patients were included in these series 92 patients**.** (Table-I).

The Ethics Committee of Tengzhou Central People’s Hospital approved the study. Informed written consent was obtained from each patient or from their relative if the patient was incapable of providing consent.

***Treatments: ***All patients received a preoperative intravenous injection of the antibiotic Ceftriaxone. General or spinal anaesthesia were used in these patients.

We used a marker (10cm) to ensure to observe the results of the plain X-rays accurate effectively. Before operation, we strictly observed the plain film using the corporation’s templates combined with the maker to determine the sizes and types of the intraoperative prosthesis and fixed the accurate Osteotomy line of femoral neck and defined the actual rotation centers. We operated carefully and cautiously and applied the method of patellar to patellar comparative test^8^, Shuck test, Drop-kick test and other methods to ensure the limbs length equality during the THA operations. Plain anteroposterior and lateral radiographs were obtained on the first postoperative day. After operation we gave these patients clear guidance of rehabilitation and physical training.

**Fig.1:**
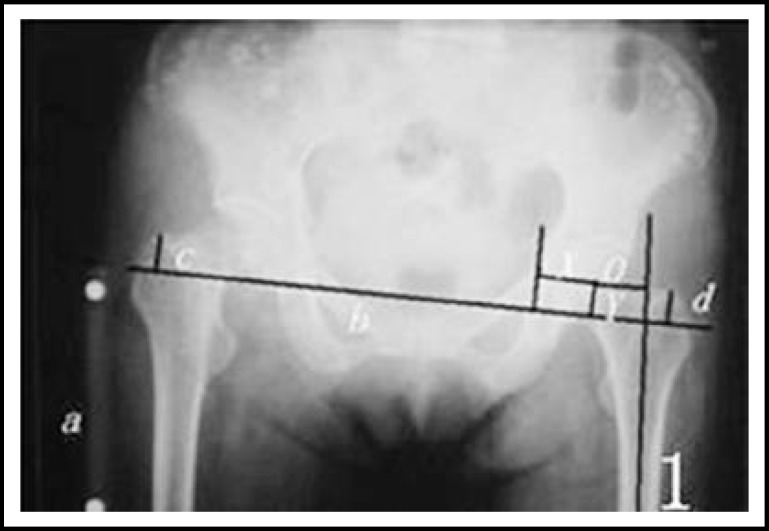
1, *a* the maker, the actual length is 10 cm 2 *b* is the line between bilateral tear-drop 3.The LLD is the difference of the length between* c* and* d* 4,*X* is the value X of rotation centers 5,*Y* is the value Y of rotation centers, the Length *Y*=*d* 6,*O* is the length of femoral offset

**Fig.2:**
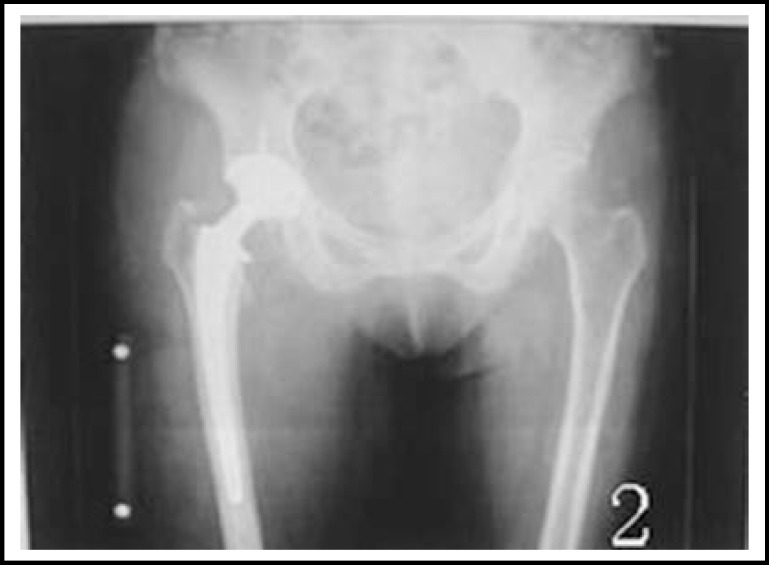
A femoral neck fracture Patient who was performed THA with hybrid prostheses. We used the marker (10cm) preoperative and used the marker or the metal or ceramic femoral head postoperative as the reference

**Fig.3:**
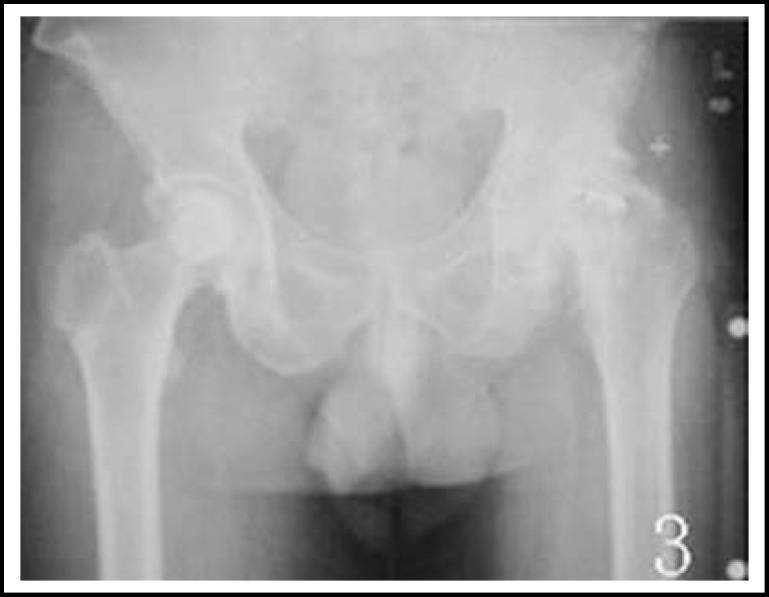
A bilateral femoral head necrosis Patient, whose left hip is very gross, but the right side is basically good and the femoral head did not depressed

**Fig.4:**
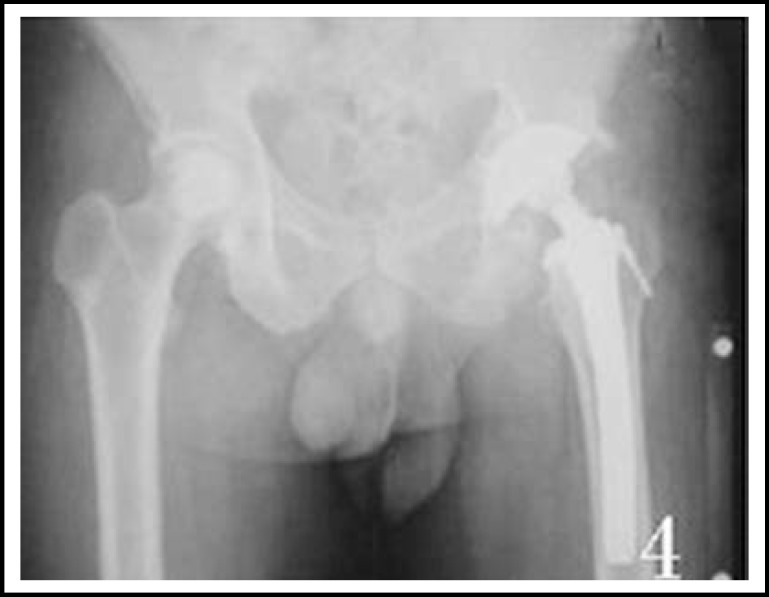
The patient was performed THA with Rebed prostheses

***Radiological measurement: ***All the plain films were taken according to the method that Lindgren^9^ introduced. The pre- and postoperative plain X-rays were studied and observed by the same doctor. The femoral offset was measured as the distance from the center of rotation of the femoral head to the long axis of the femoral shaft. The rotation centers X-value was the distance from rotation centers to the tangent line of the teardrop inner margin.^10^ The rotation centers Y-value was the distance from rotation centers to the line of bilateral teardrop. The LLD of postoperative and preoperative was the difference from the peak of the greater trochanter to the line between bilateral tear-drop. (Fig. 1, 2, 3 and 4).

***Follow-up: ***All patients had immediate postoperative anteroposterior (AP) and lateral radiographs of the hip. We took repeat radiographs yearly until the latest follow-up. No patients were lost to follow-up. The minimum follow-up was 49 months (mean, 64 months; range, 49–88 months). AP radiographs were taken with the legs positioned in 158 internal rotation with the coccyx centered 2cm above the pubic symphysis. Three of us (JAG, CAB, JM) measured all radiographs for native and reconstructed femoral offset, acetabular inclination, and acetabular ante version from the standardized radiographs using MATLAB1 (The Math-Works Inc, Natick, MA). Any interobserver difference between measurements was noted and re-measured. All patients were followed up, for from 26 months to 84 months. The statistical analysis for comparison was performed using SPSS software version 18.0 for Windows. Statistical significance of difference was determined by paired Student’s t test. A value of P<0.05 was considered significant.

## RESULTS

**Radiological result**s

***Femoral offset: ***We found that the preoperation femoral offset was 3.689±0.7538cm and the postoperation (Operated side) was 3.539±0.7271cm, p>0.05, the femoral offset was basically not changed.

**Rotation centers X, Y: **We found that the preoperation X value of rotation centers of control side was 4.14±0.801 cm, the preoperation Y value of rotation centers of control side was 2.381±0.7646 cm and the X value of rotation centers of operated side was 3.81±0.659cm, the Y value of rotation centers of operated side was 2.218±0.6979 cm the X and Y values of rotation centers decreased, p<0.05 as shown in Table-II.

**Fig.5:**
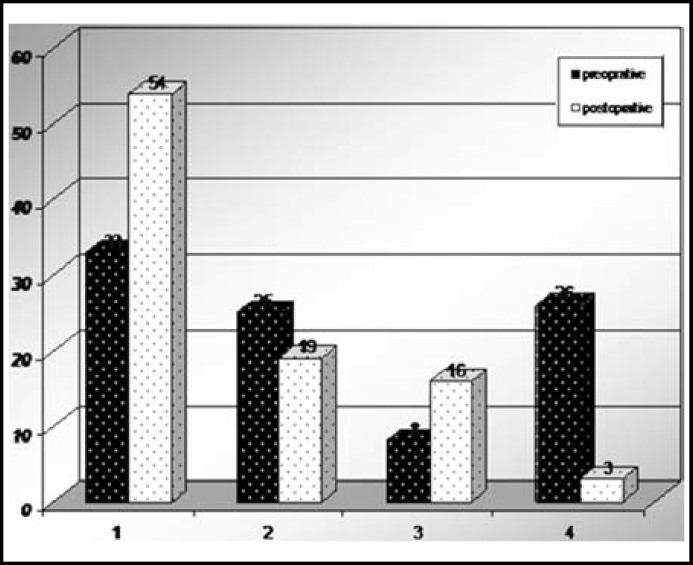
LLD, limbs length discrepancy, 1 column, LLD < 0.6cm 2 column, 1.2 cm >LLD >0.6 cm 3 column, 1.2<LLD<1.8cm 4 column, LLD > 1.8cm

***The discrepancy of two limbs: ***Before operation, we valued the LLD of all cases, the LLD of these cases were from 0 to 5.5cm. 33 0f these cases had LLD less than 0.6 cm, 25 of them with LLD more than 0.6cm but less than 1.2cm while 8 of these cases had LLD more than 1.2cm but less than 1.8cm and there were 26 cases whose LLD were more than 1.8cm. After operation, we found that the cases with LLD less than 0.6cm were 54, the cases with LLD more than 0.6cm but less than 1.2cm were 19, the cases whose LLD was more than 1.2cm but less than 1.8cm were 16 and only 3 cases had LLD over 1.8cm. The Average value of LLD was from 1.344 cm and decreased to 0.464 cm as shown in Fig.5.

***Clinical results and Complications: ***We followed up these patients form 26 months to 84 months (mean 49 months), the mean HHS of these patients were improved from 52 (31-70) to 92.3(85-96) at the latest follow-ups, no patients required a crutch to support walking. The life of these patients had a qualitative leap. We did not find any case with infection joint dislocation or any other complications.

## DISCUSSION

For total hip arthroplasty (THA), the challenge was to obtain optimal function of the reconstruction hip and to correct the femoral offset, decrease any limbs length discrepancy, and guarantee the centers of rotation of the hip joint.

**Table-I:** The patients and prosthesis in our study

*Original diagnosis*	*Sex*	*Side*	*Age*	*Prosthesis*
Osteoarthritis 33 Rheumatoid arthritis 1 Fracture neck of femur 25 Femoral head necrosis 33	Female 49 Male 43	Left 42 Right 50	Min 23 Max 78 Mean 56	Cementless 51 Cemented 27 Hybrid 14

**Table-II:** The change of pre- and postoperative femoral offset, X, Y value of rotation centre, LLD and HHS score

	*Preoperative (n=92)*	*Postoperative (n=92)*	*Statistical significance*
Femoral offset	3.689±0.7538cm	3.539±0.7271cm	NS
X value of ration center	4.14±0.801cm	3.81±0.659cm	P<0.0001
Y value of ration center	2.381±0.7646cm	2.218±0.6979cm	P=0.015
HSS	52±6.64	91±4.17	P<0.0001
LLD	0.646±0.7620 cm	1.364±1.2849cm	P<0.0001

Data are mean ± SD, Statistical significance of difference was determined by paired Student’s t test,

A value of P<0.05 was considered significant

The preservation of femoral offset was an important element of THA for reproducing the biomechanics of the hip joint after total hip arthroplasty, since it reduced the load transferred to the cup and enabled better joint stability. Inadequate femoral offset could lead to joint instability, high joint reaction forces, increased polyethylene wear, and decreased range of motion.^1^^-^^3^^,^^11^^,^^12^ For this reason, decreased femoral offset reduced abductor muscle strength, increased incidence of limp. An increase in the joint reaction force may also lead to a higher rate of wear because of decreased femoral offset. Devane PA et al.^6^ had confirmed that reduction of normal femoral offset leads to an increase in PE wear. A failure to reproduce femoral offset in THA could result in limp, fatigue, impingement, and recurrent subluxation and dislocation.^7^

After total hip arthroplasty (THA), the femoral offset was generally reduced. But our findings that the preoperative femoral offset was 3.689± 0.7646cm and postoperative (Operated side) was 3.539± 0.7271cm p>0.05, the femoral offset was basically not changed. This may be the reasons or evidences of our better clinical results.

Leg length discrepancy is also an important functional parameter that is related to success in THA. LLD was a well-known complication of THA^4^^,^^5^^,^^13^^-^^18^, Leg length inequality can contribute to low back pain, sciatic nerve palsy, ipsilateral knee pain, and abnormal force transmission across the hip joint,^14^^,^^19^^-^^23^ and may contribute to hip instability, aseptic prosthesis loosening and compromised cardiopulmonary function.^5^ Leg length inequality was also a primary cause for malpractice liability lawsuits after THA in the United States.^13^^,^^14^^,^^21^

Williamson and Reckling^23^ reported that 27% of patients required heel, Love and Wright also reported limbs lengthening of greater than 1.5 cm in 18% of the 40 patients in their reports. Several methods using pins, rulers, and calipers have been described for intraoperative correction of limbs-length inequality.^24^^-^^26^ Typically, measurement of the distance between two reference points marked on the pelvis and femur has been performed, methods using the anterior superior iliac spine or iliac wing as a reference.

 Measurement of limbs length and offset with a maker appears to be reliable, therefore eliminated the need to revise the magnification of the plain radiographs, Usually, the surgeon addressed the equalization of limbs length and restoration of offset by preoperative physical examination, manual and/or computer-assisted template, and/or the use of mechanical tools, pins and tape measures.^24^^-^^26^ In this study, we still observed data by preoperative plain film examination with the guidance of the marker, and used template of the Link corporation as a preoperative measurement tool to observe limbs length, offset and X, Y value of rotation centers and to determine the type and size of the prostheses.

In fact, all observations of the planning parameters preoperative are not fully accurate because of the magnification effect, anatomic conditions, or possible defective execution. The marker is a relatively accurate and reliable index that can be employed easily for preoperative plain film measurement during THA. So the accuracy and reliability of our observation was with relative high confidence.

Preoperative templating may be a way of restoring the required medial offset and correcting the LLD. We never denied Preoperative planning for THA enables selection of the appropriate length for the prosthetic neck and eventually the type of implants to utilize. Jasty et al^17^ had used a caliper in association with preoperative templating to measure limbs length and found that 16% of the patients had limbs length inequality after surgery Woolson et al^13^ used preoperative templating and found 86% of patients LLD less than 6mm. Several authors have described various methods to obtain correction of LLD^18^^,^^27^ with a view to restore the normal geometry of the hip during the total hip arthroplasty, Although these methods had many merits and achieved relative excellent results , there were still limitations of these technique for example, Patient's anaesthesia condition, whether Patient's body posture changed intraoperative and so on.

We used the methods from Woolson et al^13^ and Jasty et al^17^ and found that 80 percent of the patients had a postoperative LLD less than 1.2 cm, and 59% had a LLD within 6 mm. The results were not as good as theirs but we thought our results were more reliable, because first, we had performed total hip arthroplasty just only in one limbs, and the normal limbs as a reference. Second, we used marker and made our observation more accurate. Third, sometimes we had to increase the length of the neck intraoperative and accordingly to lengthen the femoral offset to maintain the hip stability. This may be a factor of our results not being so excellent.

The rotation centers had a minute lower medial shift and we did not find enough literature discussion on it. We couldn’t conclude whether the deflection of rotation centers affects the longevity of the prosthesis, but most surgeons think it is better to optimize on the reconstruction of hip^8^^,^^15^^,^^28^ because minute medial shift of rotation centers can reduce the gravitational force arm which in turn reduces the load transferred to the cup and accordingly decreases the PE wear.

There were many factors which could influence the terminal results of THA. For example, Age, femoral head size, length of follow up, femoral ante version, patient gender, weight, and activity level can affect PE wear. So our study does, however, have several limitations. Our follow-up is relatively short and therefore we are unable to guarantee the long-term advantage of the results, the number of patients was not large enough and so on. Maybe it can only reveal a part of the mechanism of total hip arthroplasty.
